# Validity and measurement invariance of abbreviated scales of the State-Trait Anxiety Inventory (STAI-Y) in a population of Italian young adults

**DOI:** 10.3389/fpsyg.2025.1443375

**Published:** 2025-02-07

**Authors:** Giuseppe Valente, Pierluigi Diotaiuti, Stefano Corrado, Beatrice Tosti, Alessandra Zanon, Stefania Mancone

**Affiliations:** Department of Human Sciences, Society and Health, University of Cassino and Southern Lazio, Cassino, Italy

**Keywords:** short anxiety scales, anxiety assessment, state anxiety, trait anxiety, psychometric validity, gender invariance, age invariance, convergent validity

## Abstract

**Introduction:**

This study evaluates the validity and measurement invariance of the 8-item scales of the State-Trait Anxiety Inventory (STAI) across gender and age. Originally developed by Spielberger (STAI-Y), these scales have been shortened to create more efficient versions without compromising psychometric robustness.

**Methods:**

A sample of Italian young adults, aged between 18 and 36 years, completed the abbreviated scales. The scales were assessed for internal consistency, convergent validity with the Endler Multidimensional Anxiety Scales (EMAS), and measurement invariance across gender and age.

**Results:**

The results demonstrated excellent internal consistency and significant correlations with the EMAS, supporting the convergent validity of the STAI-S-8 and STAI-T-8. Both scales retained balanced factorial structures and robust anxiety measurement capabilities. Measurement invariance was confirmed across gender and age, indicating the scales' reliability for anxiety assessment in young adults regardless of demographic differences.

**Discussion:**

While the findings underscore the practical utility of the 8-item STAI scales for rapid anxiety assessment in clinical and research settings, limitations include the absence of a clinical sample and reliance on self-report measures, which may introduce biases. Future research should include clinical populations and explore cultural differences in anxiety manifestation. Despite these limitations, the 8-item STAI scales offer valid, reliable, and efficient tools for measuring anxiety, with significant implications for timely interventions and enhanced psychological assessment.

## 1 Introduction

The State-Trait Anxiety Inventory (STAI) (Spielberger et al., 1971) is one of the most commonly used self-report tools for assessing anxiety in both clinical and research contexts (Evans et al., 2015; Balsamo et al., 2018; See et al., 2023; Tan et al., 2023). This instrument provides a realistic measure of two components of anxiety, differentiated as state anxiety and trait anxiety. State anxiety can be defined as an intense and unpleasant affective condition of strong apprehension but of a transitory nature. Conversely, trait anxiety can be defined as an individual characteristic related to the tendency to frequently respond with worries, agitation, and restlessness to various situations (Barros et al., 2022). Trait anxiety can be listed among the characteristic traits of an individual's personality and may be associated with various psychopathological conditions and a constant high arousal. Some research has shown that this emotional state is associated with a temporary increase in the activity of the sympathetic nervous system, but without specific pathological conditions (Spielberger and Barratt, 1972; Hoehn-Saric et al., 2004; Pacheco-Unguetti et al., 2010). However, it has not yet been clarified whether the two types of anxiety are related behaviorally or are independent characteristics.

According to an initial formulation by Spielberger (1980), anxiety can be defined as a unidimensional construct that includes both state and trait anxiety, considered different sides of the same coin (Vagg et al., 1980). Based on these two types of anxiety, the STAI was structured with two scales that measure state anxiety (STAI-S) and trait anxiety (STAI-T) respectively. In its original formulation, called STAI-X, the two scales had 20 items each. The STAI-S was composed of 20 items, half of which were positive items (anxiety-absent) and the other half were negative (anxiety-present). The original STAI-T scale, on the other hand, consisted of seven positive items (anxiety absent) and 13 negative (anxiety present). The STAI-X was revised in 1983 (Spielberger et al., 1983). In the new version, known as STAI-Y, some of the original items that were more related to depressive aspects were replaced to improve its specificity. The STAI-Y also improved the structure of the STAI-T scale by achieving a better balance of the factor structure between items related to present anxiety and those related to absent anxiety.

The Y form, the most widely used version today, includes 20 items for assessing trait anxiety and 20 for state anxiety. The state anxiety items include statements such as: “I am tense,” “I am worried,” and “I feel calm,” “I feel secure.” The trait anxiety items include: “I worry too much about something that really doesn't matter” and “I am satisfied,” “I am a stable person.” All items are rated on a 4-point scale ranging from “Almost never” to “Almost always.” Higher scores indicate a higher level of anxiety. The inventory is suitable for those with a reading level at least equivalent to elementary school graduation.

The STAI-Y is used in many clinical areas, such as alcohol use disorder (Mackus et al., 2023; Papathanasiou et al., 2023), providing normative data on anxiety (Konstantopoulou et al., 2020; Staines et al., 2022; D'Urso et al., 2023) and evaluating treatment options (Brooker, 2019; Nissen et al., 2021; Diotaiuti et al., 2021a, 2022, 2024; Tosti et al., 2024). Although the STAI-Y seems to have better psychometric properties compared to the STAI-X, both tools appear to be comparable for assessing anxiety, as the correlation between them varies from 0.96 to 0.98 (Spielberger et al., 1982).

Developing an abbreviated version of this scale can be important in research and clinical contexts to reduce administration time without reducing its reliability. Marteau and Bekker (2020) developed the first form of the STAI-S scale reduced to an abbreviated form. The researchers selected three items for present anxiety and three for absent anxiety to maintain the balance between the present and absent anxiety dimensions of the STAI-S scale. The scores of this 6-item short form are highly correlated with the full 20-item scale and are widely used in both basic research and clinical practice (Vintilescu, 2021; McGuire et al., 2022).

In many countries, short versions of the STAI scales have been developed, for example, a 6-item version from the Netherlands (van der Bij et al., 2003), a 5-item version from Japan (Koizumi et al., 1998), an 8-item version from France (Micallef et al., 1998), a 6-item version in Brazil (Fioravanti-Bastos et al., 2011), a 5-item version in Hungary (Zsido et al., 2020), a 4-item version in Spain (Buela-Casal and Guillén-Riquelme, 2017), and a 6-item version in China (Du et al., 2022). In Italy, to date, no abbreviated form of the two scales has yet been presented. Other validation efforts have focused on emergency response and psychological adjustment measures (Diotaiuti et al., 2021b).

The aim of this study was to develop a short form of the two STAI scales maintaining adequate reliability and a balance between items with present and absent anxiety in each state/trait scale in the Italian population. Attention was focused on creating a form suitable for the Italian population, through a process of item selection based on data analysis collected from representative samples of young adults. This study aims to provide a more concise but equally valid tool for assessing anxiety in various research and clinical contexts.

## 2 Methods

### 2.1 Participants

The study was conducted by recruiting two samples of participants, consisting of 746 and 431 young adults respectively. The first group was used for exploratory analyses and the second group for confirmatory analyses. Participants were recruited through announcements and invitations distributed at various Italian universities via mailing lists and postings on university bulletin boards. Additionally, recruitment campaigns were conducted on social media platforms such as Facebook and Instagram, using specific groups of interest. Data collection protocols were made available via a link on the online platform Questbase. Preliminary informed consent was collected through an online form that outlined the study's objectives, potential risks, and participation procedures, while ensuring the confidentiality of the data collected. Participation was voluntary and there was no financial compensation.

As for inclusion criteria, participants had to be between the ages of 18 and 36. This age range was chosen to focus on young adults, a demographic group often the subject of psychological studies due to its relevance in terms of personal and academic development. In order to participate in the study, individuals were required to be Italian with adequate reading and comprehension skills in the Italian language. Finally, only individuals without a prior diagnosis of psychological or neurological disorders were eligible to participate. This criterion was implemented to prevent pre-existing clinical conditions from influencing the responses to the anxiety scales, introducing potential biases into the results. Therefore, those who were using psychotropic drugs were excluded from the study as these drugs can alter emotional and cognitive responses.

The recruitment of participants in the first group took place from October to December 2023. In this group, the average age was 24.23 years, with a standard deviation of 4.47. The gender distribution showed that out of the 746 participants, 306 (41%) were male and 440 (59%) were female. The sample consisted of adults recruited predominantly from the University of Cassino and Southern Lazio. Among them, 63.6% (*n* = 474) were university students, with the following distribution by fields of study: 32% (*n* = 239) were enrolled in Engineering and Technology programs, 20% (*n* = 149) in Social Sciences and Humanities, and 11.6% (*n* = 86) in Business and Economics. The remaining 36.4% (*n* = 272) were young professionals, distributed across administrative roles (15%, *n* = 112), technical fields (12%, *n* = 89), and education (9.4%, *n* = 71). This breakdown underscores a balanced representation of university students and young professionals, offering a comprehensive view of the demographic and occupational diversity within the sample. The second group comprised 431 participants, including 184 males (42.7%) and 247 females (57.3%), with a mean age of 24 years (SD = 4.44). Of these, 68% (*n* = 293) were university students, distributed across the following fields of study: 29.8% (*n* = 128) in Social Sciences and Humanities, 25.6% (*n* = 111) in Engineering and Technology, 12.6% (*n* = 54) in Business and Economics, and 4% (*n* = 12) in other fields, such as Psychology and Biology. The remaining 32% (*n* = 138) were young professionals, categorized into administrative roles (13.9%, *n* = 60), technical fields (10%, *n* = 43), and education (8.1%, *n* = 35). This additional sample was recruited in January and February 2024, and an additional inclusion criterion was not having participated in the previous administration of the study.

### 2.2 Tools

(A) The State-Trait Anxiety Inventory—Y version (STAI-Y): (Spielberger et al., 1983; Italian trans. Pedrabissi and Santinello, 1989) is an updated version of the Spielberger Anxiety Rating Scale (STAI-X), a widely used psychometric tool for assessing anxiety in individuals. The “Y” version refers to its latest revision of 40 items, which was necessary to make the instrument more current and precise. The scale consists of two subscales: (1) the STAI-Y-S (20 items) which measures state anxiety and includes questions to assess the temporary and changing feelings of anxiety at a given moment; (2) the STAI-Y-T (20 items) which measures trait anxiety and refers to an individual's general tendency to experience anxiety as a trait of their personality. This part of the scale includes questions designed to assess a person's tendency to experience anxiety consistently over time. Responses to the questions on the STAI-Y scale provide a total anxiety score, with higher scores indicating higher levels of anxiety. This tool is widely used in clinical and research settings to assess anxiety and monitor changes over time or in response to specific treatments.

(B) The Endler Multidimensional Anxiety Scales (EMAS) is a self-assessment questionnaire composed of three easy-to-administer scales that measure different types of anxiety. The scale provides a set of measures to assess: (1) state anxiety, which is transient and situational (EMAS-S), through 20 items of which 10 measure the “emotional” component and 10 the “cognitive worry” component; (2) the individual predisposition to experience anxiety in four types of situations relative to a wide range of experiences (EMAS-T) through 60 items (15 for each proposed situation); (3) the individual perception of threat related to the situation (EMAS-P) through 5 items. The test uses a 5-point Likert scale with intensities ranging from 1 (not at all) to 5 (very much).

In addition to the psychometric scales, a demographic sheet was administered to collect data on participants' age, gender, educational background, and other relevant characteristics. The STAI-Y and EMAS scales were administered to assess state and trait anxiety across both groups. The STAI-Y State scale demonstrated excellent internal consistency, with Cronbach's α of 0.92 in Group 1 and 0.89 in Group 2. Similarly, the STAI-Y Trait scale showed Cronbach's α of 0.90 and 0.88 for Groups 1 and 2, respectively. For the EMAS scales, Cronbach's α for the EMAS-S scale was 0.88 in Group 1 and 0.85 in Group 2, while for the EMAS-T scale, it was 0.91 in Group 1 and 0.89 in Group 2.

The descriptive statistics of these scales further support their reliability and applicability in the study. For the STAI-Y State scale, Group 1 had a mean score of 45.69 (SD = 5.02) with scores ranging from 34 to 63, while Group 2 showed a mean score of 51.54 (SD = 12.11) with scores ranging from 21 to 80. For the STAI-Y Trait scale, Group 1 recorded a mean score of 46.62 (SD = 5.89) with a range of 21 to 63, while Group 2 had a mean score of 49.41 (SD = 10.43) with scores spanning from 23 to 76. For the EMAS scales, the EMAS-S scale (assessing state anxiety) demonstrated a mean score of 48.96 (SD = 20.82) in Group 1, with a range of 20 to 100, and 50.34 (SD = 20.86) in Group 2, with the same range of scores. The EMAS-T scale (assessing trait anxiety) showed a mean score of 157.87 (SD = 30.07) in Group 1, ranging from 60 to 264, and 189.26 (SD = 35.27) in Group 2, with scores spanning from 60 to 283.

### 2.3 Procedure

Two independent groups of participants were recruited. The first group (*n* = 746) completed the full version of the STAI-Y (state and trait) scales electronically, along with demographic data collection. After conducting exploratory analyses to reduce the number of scale items using this first sample, a second independent group (*n* = 431) was recruited to complete the abbreviated version of the STAI-Y (State and Trait) along with the Endler Multidimensional Anxiety Scales (EMAS). The second administration was intended for conducting confirmatory analyses and invariance testing on the abbreviated version of the STAI-Y (State and Trait). On average, participants took between 10 and 15 min to complete the questionnaire in both administrations.

## 3 Statistical analysis

The aim of the study was to obtain brief but statistically adequate forms of the STAI-S and STAI-T scales. Analyses were conducted on the first sample (*n* = 746) for the exploratory phase and on the second sample (*n* = 431) for the confirmatory analyses. The item selection procedure was based on the statistical methodology reported by Marteau and Bekker (1992). Following this procedure, the items of the STAI-S and STAI-T scales were ranked according to their “Corrected Total Item Correlation” scores. Based on this parameter, an equal number of items reflecting the presence and absence of anxiety were selected to create reduced forms of 12, 10, and 8 items for each of the STAI-S and STAI-T scales. Subsequently, the internal consistency of each of the three reduced versions was assessed by calculating their respective Cronbach's α and McDonald's ω coefficients. Pearson correlation coefficients between each of the three short forms and the full scales were calculated to evaluate the similarity between the short and full scales. A correlation value of 0.90 is generally accepted as a good indication of proportionality between the scales (Kline, 1993).

To test the adequacy of the model, a confirmatory factor analysis (CFA) was performed. As suggested by the technical literature (Teo, 2009), the indicators of model adequacy used were Chi-square, CFI (Comparative Fit Index), TLI (Tucker-Lewis Index), and RMSEA (Root-Mean-Square Error of Approximation), with CFI and TLI values > 0.95 and RMSEA < 0.06 indicating excellent model fit (Yu, 2002).

After selecting the best model for the number of items and adequacy, the model's invariance for sex and age was tested. To test age invariance, two groups were defined: 18–25 years (59.7%) and 26–36 years (40.3%).

Convergence analysis was conducted by verifying the correlation between the STAI-Short test and the EMAS test.

To establish normative data for the STAI-S-8 and STAI-T-8 scales, scores from the second sample were collected and divided by gender. For each subgroup (males, females, and the total sample), the means and standard deviations for the obtained scores were calculated. Four levels of anxiety (absent, low, moderate, and high) were defined based on the quartiles of the distributed scores. The 90th percentile was calculated to identify the upper critical scores for each subgroup. Skewness and kurtosis coefficients were calculated to assess the distribution of scores relative to normality.

## 4 Results

To select the best items for the STAI-S scale, the items were ranked based on the corrected item-total correlation coefficients (Table 1). All items showed values above the cutoff criterion of 0.3 suggested by Nunnally and Bernstein (1994), indicating a good association of these items with the total STAI-S score. Based on these corrected item-total correlation coefficients, three short versions of the STAI-S scale were created. Consequently, a 12-item scale (STAI-S-12) was composed of six items indicating the presence of anxiety and six items indicating the absence of anxiety, with the highest corrected item-total correlation coefficients (presence of anxiety items: 13, 17, 12, 6, 18, 3; absence of anxiety items: 20, 15, 5, 10, 16, 2). Subsequently, a 10-item scale (STAI-S-10) and an 8-item scale (STAI-S-8) were also formed by selecting half of the items indicating the presence of anxiety and half indicating the absence of anxiety, with the highest corrected item-total correlation coefficients and adequate fit indices.

**Table 1:** Corrected item-total correlations of the STAI-S scale (*n* = 746).

**Item**	**Nature**	***r***
**STAI-S-13**	*N*	**0.768**
**STAI-S-17**	*N*	**0.743**
**STAI-S-12**	*N*	**0.740**
**STAI-S-20**	*P*	**0.728**
**STAI-S-15**	*P*	**0.722**
**STAI-S-05**	*P*	**0.713**
**STAI-S-06**	*N*	**0.711**
**STAI-S-18**	*N*	**0.705**
**STAI-S-10**	*P*	**0.703**
**STAI-S-03**	*N*	**0.702**
STAI-S-04	*N*	0.695
STAI-S-09	*N*	0.670
**STAI-S-16**	*P*	**0.662**
**STAI-S-02**	*P*	**0.657**
STAI-S-01	*P*	0.656
STAI-S-11	*P*	0.615
STAI-S-14	*N*	0.597
STAI-S-08	*P*	0.564
STAI-S-07	*N*	0.452
STAI-S-19	*P*	0.443

Correlations in bold indicate the six higher positively (*P*) and negatively (*N*) worded items.

Table 2 presents the Cronbach's alpha and McDonald's omega coefficients for each of the three short scales with balanced positive and negative items. As expected, both Cronbach's alpha and McDonald's omega coefficients were proportional to the number of items in each of the short scales. According to these results, the STAI-S-8 had the fewest items with acceptable coefficients for both Cronbach's alpha and McDonald's omega (Nunnally and Bernstein, 1994).

**Table 2:** Cronbach's a coefficients of the STAI-S short scales (*n* = 746).

	**McDonald's ω**	**Cronbach's α**
STAI-S-12	0.925	0.924
STAI-S-10	0.918	0.917
STAI-S-8	0.893	0.892

Similarly to the STAI-S scale, the items of the STAI-T scale were also ranked based on the corrected item-total correlation coefficients. As shown in Table 3, all items except item 34 had values above the cutoff criterion of 0.3 suggested by Nunnally and Bernstein (1994), indicating a good association of these items with the total STAI-T score. Additionally, as can be seen in Table 4, all comparisons showed acceptable Cronbach's alpha coefficients, with acceptable values for both Cronbach's alpha and McDonald's omega (Nunnally and Bernstein, 1994).

**Table 3:** Corrected item-total correlations of the STAI-T scale (*n* = 746).

**Item**	**Nature**	***r***
**STAI-T-36**	***P***	**0.749**
**STAI-T-31**	***N***	**0.731**
**STAI-T-30**	***P***	**0.731**
**STAI-T-33**	***P***	**0.722**
**STAI-T-32**	***N***	**0.713**
**STAI-T-40**	***N***	**0.713**
**STAI-T-38**	***N***	**0.699**
**STAI-T-23**	***P***	**0.693**
**STAI-T-28**	***N***	**0.690**
**STAI-T-21**	***P***	**0.687**
**STAI-T-27**	***P***	**0.680**
**STAI-T-37**	***N***	**0.670**
STAI-T-35	*N*	0.666
STAI-T-22	*N*	0.614
STAI-T-25	*N*	0.602
STAI-T-29	*N*	0.598
STAI-T-26	*P*	0.518
STAI-T-34	*P*	0.451
STAI-T-39	*P*	0.392
STAI-T-24	*P*	−0.493

Correlations in bold indicate the six higher positively (*P*) and negatively (*N*) worded items.

**Table 4:** Cronbach's a coefficients of the STAI-T short scales (*n* = 746).

	**McDonald's ω**	**Cronbach's α**
STAI-T-12	0.911	0.912
STAI-T-10	0.900	0.899
STAI-T-8	0.875	0.874

The factor structure of the STAI-S-8 scale was evaluated using a factor analysis with Principal Axis Factoring and Varimax rotation. The analysis of the eigenvalues and the Scree plot, along with the interpretation of the factors, indicated a two-factor solution. The rotated factor loadings for this two-factor solution are presented in Table 5. The first factor accounted for 29.3% of the variance and is consistent with the interpretation of anxiety-present, incorporating all four items related to this construct. The second factor explained 27.8% of the variance and is consistent with the interpretation of anxiety-absent, including all four items associated with this construct. In total, the model explains a cumulative variance percentage of 57.1%. The KMO value for the STAI-T-8 scale was 0.915, indicating a high model adequacy.

**Table 5:** Factor loadings (structure matrix) (*n* = 746).

	**Factor 1**	**Factor 2**
STAI-S-05	0.736	
STAI-S-15	0.706	
STAI-S-02	0.598	
STAI-S-10	0.597	
STAI-S-06		0.697
STAI-S-17		0.696
STAI-S-12		0.664
STAI-S-03		0.625

Applied rotation method is varimax.

The factor structure of the STAI-T-8 scale was also evaluated using a Principal Component Analysis with Varimax rotation. Similarly, the analysis of the eigenvalues and the Scree plot, along with the interpretation of the factors, indicated a two-factor solution. Table 6 presents the rotated factor loadings for a two-factor solution. A well-defined two-factor structure (anxiety-present and anxiety-absent) was found. The first factor accounted for 28.5% of the variance. The second factor explained 28.2% of the variance. In total, the model explains a cumulative variance percentage of 56.7%. The KMO value for the STAI-T-8 scale was 0.894, indicating a high model adequacy.

**Table 6:** Factor loadings (structure matrix) (*n* = 746).

	**Factor 1**	**Factor 2**
STAI-T-40	0.729	
STAI-T-38	0.701	
STAI-T-31	0.699	
STAI-T-28	0.643	
STAI-T-33		0.769
STAI-T-23		0.696
STAI-T-27		0.667
STAI-T-21		0.626

Applied rotation method is varimax.

The second sample of participants was used to conduct the confirmatory factor analysis (CFA) of the STAI-S-8 and STAI-T-8 scales, with the aim of verifying the factor structure of the anxiety scales. The data collected from the administrations were analyzed using AMOS 20.0 software to conduct a confirmatory factor analysis (CFA) on the reduced version of the instrument. Path coefficients were estimated and tested to evaluate the model fit, according to a standard defined in previous research (Wu, 2013). The confirmatory factor analysis, commonly used to test the consistency between the observed covariance matrix in the sample and the estimated one, provided the following fit indices for the STAI-S-8: *X*^2^/*df* = 2.845, RMSEA = 0.050 (90% CI, 0.034–0.066), CFI = 0.987, and TLI = 0.982; while for the STAI-T-8, the results were: *X*^2^/*df* = 2.398, RMSEA = 0.043 (90% CI, 0.027–0.060), CFI = 0.990, and TLI = 0.985 (see Table 7).

**Table 7:** Confirmatory factor analysis (CFA) of the short-form STAI scales (*n* = 431).

**Index**	**STAI-S-8**	**STAI-T-8**
	**Value**	**Value**
*X*^2^/*df*	2.845	2.398
RMSEA	0.050	0.043
RMSEA IC 90%	0.034–0.066	0.027–0.060
Comparative fit index (CFI)	0.987	0.990
Tucker-Lewis index (TLI)	0.982	0.985
Bentler-Bonett normed fit index (NFI)	0.981	0.982
Parsimony normed fit index (PNFI)	0.666	0.667
Bollen's relative fit index (RFI)	0.972	0.974
Bollen's incremental fit index (IFI)	0.988	0.990

Following the confirmatory analysis of the model, convergent validity was tested through a correlational analysis with the EMAS-S and EMAS-T scales (The Endler Multidimensional Anxiety Scales; Endler et al., 1991), which respectively assess state anxiety and trait anxiety. In Table 8, a significant correlation can be observed between the mean scores of the STAI-S (8 items) and the EMAS-S (Total), indicated by a Pearson correlation coefficient (*r*) of 0.773, *p* < 0.001. Additionally, there were also highly positive correlations between the mean scores of the STAI-T (8 items) and the EMAS-T (Total), with a Pearson correlation coefficient (*r*) of 0.691, *p* < 0.001.

**Table 8:** Pearson's correlations (*n* = 431).

	**STAI-S-8**	**STAI-T-8**	**EMAS-S**	**EMAS-T**
STAI-S-8	1			
STAI-T-8	0.687^**^	1		
EMAS-S	0.773^**^	0.693^**^	1	
EMAS-T	0.599^**^	0.691^**^	0.662^**^	1

^**^Correlation is significant at the 0.01 level (2-tailed).

These results suggest a good concordance between the 8-item State and Trait Anxiety Scales and the Endler Multidimensional Anxiety Scales (EMAS) S and T, thereby supporting the convergent validity of the scales used in the present study.

Furthermore, we conducted an analysis to assess the invariance of the factor structure of the STAI-S-8 and STAI-T-8 scales in relation to gender and age. By examining four nested models characterized by different levels of restriction, we tested invariance for gender and age. Tables 9, 10 meticulously report the results with the goodness-of-fit indices for the model, considering gender variables and the nested invariance models. These tables are organized based on the progressive level of restriction, for both the STAI-S-8 and STAI-T-8 scales. Based on the results obtained, significant gender invariance emerged for both scales. The adequacy of the model fit for males and females was evaluated as good. These important findings suggest that latent means can be appropriately compared based on gender.

**Table 9:** Tested models and goodness-of-fit indices STAI-S-8 (*n* = 431).

	**χ^2^**	***df***	***Δχ* ^2^**	**Δ*df***	**CFI**	**TLI**	**RMSEA**	**ΔCFI**	**ΔTLI**	**ΔRMSEA**
**Models in each group**										
**Gender**										
Male	40.130	19			0.980	0.971	0.060			
Female	48.230	19			0.983	0.975	0.059			
**Gender**										
Configural	88.362	38	–	–	0.982	0.973	0.060	–	–	–
Metric	92.053	44	3.69	6	0.983	0.978	0.054	0.001	0.005	−0.006
Scalar	101.573	50	9.523	6	0.981	0.979	0.053	−0.002	0.001	−0.001
Strict	103.986	58	2.413	8	0.983	0.984	0.046	0.002	0.005	−0.007

*df* , degrees of freedom; χ^**2**^, Chi square; Δχ^**2**^, difference in Chi square; Δ*df* , difference in degrees of freedom; *CFI*, comparative fit index; *TLI*, Tucker-Lewis index; *RMSEA*, root mean square error of approximation; Δ*CFI*, difference in comparative fit index; Δ*TLI*, difference in Tucker-Lewis index; Δ*RMSEA*, difference in root mean square error of approximation.

**Table 10:** Tested models and goodness-of-fit indices STAI-T-8 (*n* = 431).

	**χ^2^**	***df***	***Δχ* ^2^**	**Δ*df***	**CFI**	**TLI**	**RMSEA**	**ΔCFI**	**ΔTLI**	**ΔRMSEA**
**Models in each group**										
**Gender**										
Male	43.188	19			0.978	0.967	0.065			
Female	21.562	19			0.998	0.997	0.018			
**Gender**										
Configural	64.750	38	–	–	0.989	0.984	0.043	–	–	–
Metric	68.775	44	4.025	6	0.990	0.988	0.039	0.001	0.004	−0.004
Scalar	85.112	50	16.337	6	0.986	0.984	0.043	−0.004	−0.004	0.004
Strict	96.779	58	11.667	8	0.985	0.985	0.042	−0.001	0.001	−0.001

*df* , degrees of freedom; χ^**2**^, Chi square; Δχ^**2**^, difference in Chi square; Δ*df* , difference in degrees of freedom; *CFI*, comparative fit index; *TLI*, Tucker-Lewis index; *RMSEA*, root mean square error of approximation; Δ*CFI*, difference in comparative fit index; Δ*TLI*, difference in Tucker-Lewis index; Δ*RMSEA*, difference in root mean square error of approximation.

Tables 11, 12 show the results of the invariance analyses for the two age groups 18–25 years and 26–36 years. Here again, the model is stable and invariant across the different nested models with increasing levels of restriction. These results indicate that latent means can also be compared based on age.

**Table 11:** Tested models and goodness-of-fit indices STAI-S-8 (*n* = 431).

	**χ^2^**	***df***	***Δχ* ^2^**	**Δ*df***	**CFI**	**TLI**	**RMSEA**	**ΔCFI**	**ΔTLI**	**ΔRMSEA**
**Models in each group**										
**Age**										
18–25 years old	54.060	19			0.987	0.982	0.050			
26–36 years old	34.724	19			0.979	0.969	0.066			
**Age**										
Configural	75.438	38	–	–	0.987	0.980	0.051	–	–	–
Metric	76.759	44	1.321	6	0.988	0.985	0.045	0.001	0.005	−0.006
Scalar	86.756	50	9.997	6	0.987	0.985	0.044	−0.001	0.000	−0.001
Strict	96.609	58	9.853	8	0.986	0.987	0.042	−0.001	−0.001	−0.002

*df* , degrees of freedom; χ^**2**^, Chi square; Δχ^**2**^, difference in Chi square; Δ*df* , difference in degrees of freedom; *CFI*, comparative fit index; *TLI*, Tucker-Lewis index; *RMSEA*, root mean square error of approximation; Δ*CFI*, difference in comparative fit index; Δ*TLI*, difference in Tucker-Lewis index; Δ*RMSEA*, difference in root mean square error of approximation.

**Table 12:** Tested models and goodness-of-fit indices STAI-T-8 (*n* = 431).

	**χ^2^**	***df***	***Δχ* ^2^**	**Δ*df***	**CFI**	**TLI**	**RMSEA**	**ΔCFI**	**ΔTLI**	**ΔRMSEA**
**Models in each group**										
**Age**										
18–25 years old	45.576	19			0.990	0.985	0.043			
26–36 years old	28.837	19			0.986	0.980	0.052			
**Age**										
Configural	66.903	38	–	–	0.989	0.984	0.045	–	–	–
Metric	73.192	44	6.289	6	0.989	0.986	0.042	0.000	−0.002	−0.003
Scalar	85.358	50	12.166	6	0.986	0.985	0.044	−0.003	−0.001	0.002
Strict	96.376	58	11.018	8	0.985	0.986	0.042	−0.001	0.001	−0.002

*df* , degrees of freedom; χ^**2**^, Chi square; Δχ^**2**^, difference in Chi square; Δ*df* , difference in degrees of freedom; *CFI*, comparative fit index; *TLI*, Tucker-Lewis index; *RMSEA*, root mean square error of approximation; Δ*CFI*, difference in comparative fit index; Δ*TLI*, difference in Tucker-Lewis index; Δ*RMSEA*, difference in root mean square error of approximation.

In Table 13, the mean scores and standard deviations for the total sample and the groups tested in the previous analysis are reported. The scores are divided into items with anxiety-present and anxiety-absent (summed in reverse).

**Table 13:** Mean and standard deviation for total sample (*N* = 431), males (*n* = 184), females (*n* = 247), and age groups (18–25 years: *n* = 261; 26–36 years: *n* = 170).

	**Total**		**Males**		**Females**		**Age 18–25**		**Age 26–36**	
	**Mean**	**DS**	**Mean**	**DS**	**Mean**	**DS**	**Mean**	**DS**	**Mean**	**DS**
STAI-S-8 Anxiety Present	9.52	2.93	9.15	2.71	10	3.25	9.74	2.97	9.48	3.18
STAI-S-8 Anxiety Absent	8.93	2.69	9.04	2.69	8.29	2.68	8.51	2.62	8.76	2.84
STAI-T-8 Anxiety Present	9.72	3.09	9.38	3.23	10.27	3.05	9.95	3.18	9.80	3.12
STAI-T-8 Anxiety Absent	9.73	2.74	9.80	2.70	9.18	2.69	9.43	2.64	9.46	2.82

The data for males and females were processed by dividing them into quartiles to distinguish between absence of anxiety, low anxiety, medium anxiety, and high anxiety. Additionally, the 90th percentile was calculated to identify the category associated with anxiety disorders. Table 14 presents these data systematically.

**Table 14:** Normative data (four anxiety levels, 90th percentile, means, standard deviation, Skewness and Kurtosis) of the STAI-S-8 and STAI-T-8 for males (*n* = 184), females (*n* = 247), and total sample (*n* = 431).

	**Absent**	**Low**	**Medium**	**High**	**90°Perc**	**M**	**DS**	**Skewness**	**Kurtosis**
**Total sample**									
STAI-S-8	8–17	18–21	22–25	26–32	29–32	21.02	5.32	−0.079	−0.538
STAI-T-8	8–17	18–20	21–24	25–32	29–32	20.44	5.23	0.088	−0.504
**Males**									
STAI-S-8	8–16	17–20	21–23	24–32	28–32	20.11	4.94	−0.116	−0.530
STAI-T-8	8–16	17–19	20–23	24–32	28–32	19.58	5.24	0.255	−0.319
**Females**									
STAI-S-8	8–18	19–22	23–26	27–32	29–32	21.70	5.50	−0.134	−0.575
STAI-T-8	8–17	18–21	22–25	26–32	28–32	21.09	5.14	−0.022	−0.528

Through further descriptive analysis, an observation emerged regarding the two groups. At the 90th percentile, women more frequently fell into the range associated with anxiety disorders compared to the male sample. These findings align with previous studies on gender differences in anxiety during stressful periods, such as the COVID-19 lockdown (Galli et al., 2022). Specifically, for state anxiety, 13.4% of women exceeded this critical level, while for trait anxiety this percentage was 9.3%. These values are higher than those recorded in the male sample, where only 4.3 and 6.5% exceeded the 90th percentile for state and trait anxiety, respectively.

## 5 Discussion

This study represents a first attempt to develop and validate an abbreviated version of the STAI (Tables 15, 16), adapted for the Italian context. The results indicate that both the STAI-S and STAI-T can be reduced to scales of only 8 items without compromising their psychometric qualities. The STAI-S-8 demonstrated good internal consistency and a balanced structure, with items assessing both the presence and absence of anxiety as in the original formulation. These observations are in line with findings from previous studies validating shortened versions of the STAI-S in other countries (van der Bij et al., 2003; Micallef et al., 1998; Fioravanti-Bastos et al., 2011; Zsido et al., 2020; Du et al., 2022). These results were also confirmed for the abbreviated version of the STAI-T-8 scale, which showed a satisfactory internal consistency coefficient and a clearly defined factor structure, with items representing both the presence and absence of trait anxiety.

**Table 15:** STAI-S short.

**Item N**	**Italian text**	**English translation**	**Anxiety type**
2	Mi sento sicuro	I feel secure	Anxiety-absent
3	Sono teso	I feel tense	Anxiety-present
5	Mi sento tranquillo	I feel calm	Anxiety-absent
6	Mi sento turbato	I feel upset	Anxiety-present
10	Mi sento a mio agio	I feel at ease	Anxiety-absent
12	Mi sento nervoso	I feel nervous	Anxiety-present
15	Sono rilassato	I feel relaxed	Anxiety-absent
17	Sono preoccupato	I feel worried	Anxiety-present

**Table 16:** STAI-T short.

**Item N**	**Italian text**	**English translation**	**Anxiety type**
21	Mi sento bene	I feel good	Anxiety-Absent
23	Sono soddisfatto di me stesso	I feel satisfied with myself	Anxiety-Absent
27	Io sono calmo, tranquillo e padrone di me	I feel calm, tranquil, and in control	Anxiety-Absent
28	Sento che le difficoltà si accumulano tanto da non potere superare	I feel overwhelmed by difficulties I cannot overcome	Anxiety-Present
31	Mi vengono pensieri negativi	I have negative thoughts	Anxiety-Present
33	Mi sento sicuro	I feel secure	Anxiety-Absent
38	Vivo le delusioni con tanta partecipazione da non potermene togliere dalla testa	I dwell on disappointments and can't get them out of my mind	Anxiety-Present
40	Divento teso e turbato quando penso alle mie attuali condizioni	I become tense and upset when thinking about my current situation	Anxiety-Present

The analyses from this study highlighted a strong convergent validity between the 8-item State and Trait Anxiety Scales (STAI) and the Endler Multidimensional Anxiety Scales (EMAS) State and Trait. This validity was observed for both the assessment of state anxiety and trait anxiety. The significant correlation between the scores obtained on the 8-item STAI State and Trait scales and the EMAS State and Trait (Total) indicates that the abbreviated Anxiety Scales can reliably and validly capture the overall levels of anxiety experienced by participants. This suggests that the abbreviation of the STAI scales did not compromise their ability to accurately assess anxiety compared to the full version of the EMAS-S in a sample of Italian adults. The possibility of shortening both STAI scales to eight items while maintaining their psychometric properties intact represents a valuable option for both research and clinical settings.

Despite efforts to conduct a thorough and comprehensive study, several limitations have emerged. One of the main limitations of our study concerns the sample composition, which was limited to young adults aged 18 to 36. Although this allowed for a detailed exploration of anxiety experiences in this specific age group, it may have limited the generalizability of our results to other age groups. During the study, it emerged that an important limitation concerns the use of self-report scales to assess anxiety levels. Self-report scales rely on participants' subjective responses and may be influenced by various factors, such as individual perception, mood, or the tendency to provide socially desirable responses (Kreitchmann et al., 2019). For example, affective dependence can play a significant role in subjective reporting (Petruccelli et al., 2014). A brief instrument is even more susceptible to potential manipulation by the patient (Hardman et al., 2022). However, despite these limitations, self-report scales are a widely used and validated tool for assessing anxiety in various research and clinical contexts. We took appropriate measures to mitigate the risk of response bias, such as ensuring anonymity of responses and providing clear instructions to participants on how to complete the scales. Nonetheless, it is important to interpret our results carefully considering the possible impact of limitations associated with the use of self-report scales.

The lack of a clinical sample represents a significant limitation of our study. Although our results confirm the validity of the 8-item STAI in assessing anxiety in young adults, their applicability in clinical settings remains to be explored. The lack of participants diagnosed with anxiety disorders may have limited our understanding of the ability of the abbreviated STAI to distinguish between normal and pathological anxiety levels. Future studies should include a clinical sample to examine more thoroughly the effectiveness of the 8-item STAI in detecting and assessing anxiety disorders and to provide empirical support for their utility in clinical practice.

For future research, it may be interesting to further explore the invariance of gender and age in other age groups and different populations and stress-related activities to validate these findings. For instance, choking episodes in archery have been explored as a specific stress condition (Diotaiuti et al., 2021c). Additionally, possible cultural differences in the experience and manifestation of anxiety could be examined to develop even more valid and culturally sensitive measures.

The practical implications of this study are significant, as the abbreviated STAI scales can be used as screening tools in various contexts where a quick and accurate analysis of anxiety symptoms is needed (Du et al., 2022). This could help to promptly identify individuals with high levels of anxiety, allowing for early and targeted interventions. Furthermore, the inclusion of the 8-item STAI in assessment protocols with other scales could be particularly advantageous in studies requiring a detailed and multi-dimensional evaluation of subjects, such as clinical studies, psychological research, or studies on the effectiveness of treatments. In the research field, integrating the 8-item STAI with other relevant scales allows researchers to gain a more comprehensive understanding of participants' psychological conditions and potential relationships between anxiety and other disorders or psychological variables. The use of the 8-item STAI along with other assessment scales can also facilitate the comparability of results between different studies and allow for better standardization of psychological assessment. This can be particularly important for developing diagnostic guidelines or assessing the effectiveness of therapeutic interventions.

## 6 Conclusions

In conclusion, this study examined the validity of the 8-item State and Trait Anxiety Scales (STAI) as well as the gender and age invariance in the use of these scales. The results confirmed the robustness of the 8-item STAI in assessing anxiety in young adults, maintaining their validity and reliability regardless of the gender and age of the participants.

The gender and age invariance in the 8-item STAI suggests that these scales can be reliably and validly used to assess anxiety in both men and women, as well as in different age groups ranging from 18 to 36 years. These results are crucial to ensure that anxiety measures are equally valid and applicable to all individuals, regardless of gender or age differences.

The abbreviated scales offer several advantages. For example, they tend to reduce response bias, a phenomenon more common in longer and more time-consuming scales (Ackerman et al., 2010). The shortened versions are much more practical, especially in clinical settings where time pressure makes it difficult to use full scales, especially when included in protocols with other assessment scales (Beidas et al., 2015).

The inclusion of gender and age invariance in the analysis of the 8-item STAI enriches our understanding of the validity and applicability of these scales, offering significant opportunities for future research and clinical practice in the field of mental health.

## Data Availability

The raw data supporting the conclusions of this article will be made available by the authors, without undue reservation.
